# Updates to the EU Directives on Professional Qualifications must align with global standards

**DOI:** 10.1371/journal.pgph.0007272

**Published:** 2026-09-21

**Authors:** Lia Brigante, Anna af Ugglas, Daniela Drandic, Jacqueline Dunkley Bent, Margrieta Langins, Mervi Jokinen, Kerry Phillips

**Affiliations:** 1 International Confederation of Midwives, The Hague, Netherlands; 2 World Health Organization Regional Office for Europe, Copenhagen, Denmark; 3 European Forum of National Nursing and Midwifery Associations, London, United Kingdom; 4 World Health Organization Collaborating Centre for Midwifery Development, Cardiff, United Kingdom; Jawaharlal Nehru Medical College, INDIA

## Abstract

The European Union (EU) Directive on Minimum Professional Qualifications: Midwife is under review as part of an ongoing regulatory update. This revision presents a critical opportunity to modernise the regulation of midwifery education and practice across the EU and EEA, having implications that extend beyond the region, given the EU’s role in influencing professional regulation globally. We argue that updates to the Directive must be aligned with current scientific evidence, Global Standards for the profession, and relevant World Health Organization guidelines. Aligning the Directive with these evidence-based standards is essential to ensure high-quality care, enable midwives to practise to their full scope, support workforce mobility, and strengthen health systems across Europe.

## Introduction

Regularly updated international standards for education, regulation and scope of practice of health professionals are one of the key ways to ensure quality, accountability and free movement of health workers globally, while improving outcomes for women and newborns [1]. However, legislative and regulatory frameworks do not always evolve at the same pace as international evidence and professional standards. In the European Union (EU), the Directive on Minimum Professional Qualifications sets the minimum standards for education for seven regulated professions, including midwives.

This Directive that regulates midwives (2005/36/EC) is currently being updated for the first time in 21 years. We argue that updates to the Directive should be aligned with international standards. Although this Directive applies formally to EU and EEA Member States, the implications of this extend the region. The EU often functions as a global norm-setter in regulation. Moreover, its standards are usually referenced in policy reform processes in other countries across different regions. In that sense, the current update represents more than a regional regulatory update; it is also a significant moment in global health policy.

## Background

Globally, sexual, reproductive, maternal, newborn and adolescent health (SRMNAH) confronts significant challenges. These include persistent inequalities in accessing proper care, and a shortage and uneven distribution of the SRMNAH workforce. Reinforcing Primary Health Care (PHC) is a vital part of the solution and midwives play a central role within PHC systems, as they are able to provide around 90% of essential SRMNAH services, when educated, regulated and enabled to practise to international standards. However, achieving this potential depends on workforce availability, but also on regulatory and educational frameworks that ensure that midwives are prepared to deliver evidence-based care throughout their full scope of practice. Education and regulatory frameworks are therefore critical components of health system strengthening, influencing the quality of care, workforce mobility, and the capacity of health systems to respond to evolving population health needs.

While international standards for midwifery education and practice have evolved considerably over the past two decades, legislative and regulatory frameworks have not kept the pace. Therefore, a growing gap between contemporary evidence-based standards and legal binding professional requirements is in place. The ongoing revision of the European Union (EU) Directive on Minimum Professional Qualifications for Midwives provides a timely case study of this broader global health policy challenge. Although the Directive formally applies to EU and European Economic Area (EEA) Member States, its influence extends well beyond Europe, as EU regulatory frameworks frequently inform education, professional regulation and workforce reforms internationally. Aligning regional legislation with evolving global standards is therefore relevant not only for Europe, but also for broader discussions on health workforce development and global standard-setting.

Several global and regional stakeholders create standards for education, regulation and scope of practice for midwives. In Europe, the essential stakeholders are the World Health Organization (WHO), International Confederation of Midwives (ICM) and European Commission (EC). These organisations have complementary but distinct roles: WHO develops normative health guidance, ICM establishes globally endorsed professional standards and competencies, and the European Commission sets the legally binding minimum professional qualifications for automatic recognition across the EU. Understanding these distinct but interconnected roles helps explain why alignment between international standards and regional legislation is central to the policy recommendations presented in this paper.

### The World Health Organization – Global and European region

The WHO regularly issues guidelines for SRMNAH services, including for antenatal, intrapartum and postnatal care [2]. Most recently, the WHO has issued two key documents: Transitioning to midwifery models of care: global position paper [3], and Implementation guidance on transitioning to midwifery models of care [4], as well as the Compendium on Respectful Maternal and Newborn Care [5].

In the European Region, WHO has been advocating for standards in midwifery education since as early as 2000 [6], when the Munich Declaration was signed by 48 countries across the region, committing them to developing common regulatory frameworks that would enable midwives “to work to their full potential both as independent and as interdependent professionals” [7]. WHO Europe regularly assessed progress [8], encouraging countries to continue to align their legislation with WHO standards and guidelines.

WHO Europe continues to advocate for countries to align with ICM Global Standards for Midwifery Education, Essential Competencies for Midwifery Practice and Global Standards for Regulation. This alignment was reiterated in the WHO/UNFPA State of the Midwifery Workforce Report [9] and the Report on Midwifery Education in Eastern Europe and Central Asia [10]. Both publications build on the ICM Global Standards for Midwifery Education and the Essential Competencies for Midwifery Practice [11,12]. The most recent Roadmap to guide implementation of the Global Strategic Directions for Nursing and Midwifery in the WHO European Region reiterates the need to align midwifery education with international standards. Specifically, they state that by 2025, WHO European Region Member States “should ensure that midwifery education benefits from alignment with the European Professional Qualification Directive, while midwifery education is guided by the International Confederation of Midwives Global Standards for Midwifery Education” [13]. In addition, WHO European Region has developed tools such as the Midwifery Assessment Tool for Education (MATE), which provides Member States with an evidence-based framework and self-assessment approach to support the strengthening of midwifery education programmes [14].

Together, these initiatives establish a clear expectation that national and regional regulatory frameworks should remain aligned with internationally recognised, evidence-based standards.

### The International Confederation of Midwives

Since 1902, the International Confederation of Midwives (ICM) has brought together midwives’ associations globally. The organisation sets the Global Standards for Midwifery Education [11], Global Standards for Midwifery Regulation [15], and scope of practice through the Essential Competencies for Midwifery Practice (the Essential Competencies) [12].

The Global Standards and Essential Competencies are updated every five years to ensure that they reflect the most robust and recent empirical and scientific evidence. The most important of these for updates to the EU Directive is the Essential Competencies for Midwifery Practice, which were updated in 2024. The process has been described in detail in the literature [16], with the process including ICM midwives associations, the ICM Board, educators, regulators, practitioners from all ICM Regions, UN agencies and global health experts, harmonised with the most recent global guidelines by organisations such as the WHO and UNFPA. The final version was voted on by the ICM Council, made up of delegates from each of ICM’s 117 member associations, which includes 31 countries in the EU / EEA. The vote to endorse the Essential Competencies for Midwifery Practice in 2024 was unanimous (100%), demonstrating a very strong global consensus for their content. However, many of these updated competencies are not yet reflected in the current EU Directive, highlighting the growing gap between evolving international standards and existing legislative frameworks.

### European Commission

In the 1970s and 1980s, the European Commission prepared separate sectoral directives for the regulated professions, developed by advisory committees. These committees agreed the minimum standards for each regulated profession, with the goal of ensuring that qualifications could be mutually recognised by all Member States, defining minimum content and length of education programmes. The purpose of these was to ensure freedom of movement in the EU + EEA, for EU + EEA nationals to be able to practice their professions in other Member States. The Directive for midwifery, Directive 80/155/EEC was passed in 1980 [17]. In 2013, the European Parliament and Council published Directive 2013/55/EU [18], amending previous Directive 2005/36/EC. The changes aimed to enhance professional mobility and standards across Member States. Key updates included granting the Commission the delegated power to review and modify training requirements for sectoral professions listed in Annex V of the Directive, including midwives. Member States were also required to strengthen continuous professional development, particularly for professions under automatic recognition, such as midwives. While amendments to Articles 40, 41, and 43 concerning midwives were enacted, updates to Annex V points 5.51 and 5.5.2 were postponed for future action by the Commission. The current update therefore, is the first update since 2005.

The creation of the European Education Area through the Bologna Process in 1999 further standardised education across the EU / EEA by creating a common framework for degrees in higher education, and a common European Credit Transfer and Accumulation System (ECTS). This allows for free movement of midwifery researchers, educators and students across the EU.

Updates to the EU Directive to date have included engagement from midwifery stakeholders [19], but overall, the current iteration of the Directive is not aligned with current ICM Global Standards for the profession [20].

As the European Commission is responsible for establishing legally binding minimum professional qualifications, the current revision represents a critical opportunity to align regional legislation with internationally agreed standards and contemporary evidence.

## Aligning the Directive with International Standards

The current revision of the Directive presents a unique policy window to strengthen coherence between regional legislation and internationally recognised standards. This alignment seeks to ensure that the minimum requirements underpinning automatic recognition reflect contemporary scientific evidence, WHO guidance and internationally endorsed professional competencies. Because the EU Directive also influences regulatory reforms beyond Europe, these considerations have broader implications for global health workforce development and governance.

Importantly, alignment does not imply harmonising all aspects of national midwifery education or limiting Member States that already exceed international minimum standards. Rather, it aims to ensure that the minimum requirements underpinning automatic recognition are consistent with contemporary evidence and internationally endorsed professional standards, while allowing countries to maintain or adopt higher national requirements according to their health system needs and contexts.

Updates to the Directive on Minimum Professional Qualifications: Midwife and all legislation regarding midwifery practice, and health policy more broadly, should be aligned with ICM Global Standards for Midwifery Education, Global Standards for Midwifery Regulation, and Essential Competencies for Midwifery Practice, as well as WHO guidelines for a number of key reasons. The key policy recommendations for updates to the Directive are summarised in Box 1. The rationale for these recommendations is outlined below:

1. **Evidence-based practice and global consensus**

ICM’s Global Standards and Essential Competencies reflect the latest research, best practice, the need for greater advocacy in the SRMNAH field and are aligned with WHO standards [21]. Alignment ensures midwives are educated and regulated to provide a consistent scope of practice, consult and refer to other professions when necessary. This in turn improves outcomes for women and newborns.

The Essential Competencies are updated to reflect the most recent scientific evidence and align with SRMNAH stakeholders every five years, through a global consultation process. In their final form they are approved by ICM’s Council, representing over 113 professional associations globally, reflecting a global consensus. Aligning the Directive with these standards would help ensure that the minimum educational requirements remain evidence-based while reflecting broad international professional consensus.

2. **Ensuring consistency across borders**

The EU Directive on Professional Qualifications was designed to facilitate the free movement of workers. Yet, considerable gaps in midwifery education across EU Member States create inconsistent standards for entry to the profession making it impossible for some professionals to practice in other EU countries [22,23]. Updating the Directive would address these gaps and improve the ethical mobility and migration of workers within the EU [24].

Beyond mobility within the EU, clearer alignment between regional legislation and internationally recognised competencies may also support more transparent and consistent assessment of qualifications obtained outside the EU, in line with the principle of the WHO Global Code of Practice on the International Recruitment of Health Personnel [25].

3. **Accountability to the Public and Taxpayers**

Legislation based on Global Standards helps ensure public confidence in health services, ensures ethical practice and accountability.

Aligning the Directive with current evidence and international standards promotes greater consistency in midwifery practice across the EU, helping to ensure that women, families and the public can expect the same level of care regardless of where they receive it.

Furthermore, many EU countries have contributed official development assistance (ODA) for global organisations like ICM, WHO and UNFPA to develop Global Standards and guidelines. A fiscally responsible and accountable approach to policy updates in the EU should therefore reflect the Standards and guidelines that European taxpayers have already invested in. Alignment also reinforces the credibility of the EU as a global development partner. Ensuring that EU legislation reflects these same standards demonstrates policy coherence between domestic regulation and international development commitments.

4. **Improving Health System Efficiency and Resilience**

Clarifying and aligning the EU Minimum Professional Standards for Midwives with Global Standards aids workforce optimisation, avoiding duplication of services, improves access, including for vulnerable groups, and ensures health system resources are used more efficiently.

Additionally, midwives are essential providers of sexual, reproductive, maternal and newborn care during humanitarian crises, disasters and climate-related emergencies. Preparing the workforce to respond safely and effectively in these contexts requires education that reflects contemporary evidence and emerging health system needs. This also contributes to health system resilience, ensuring that midwives have the education needed to address national population health needs and future service models [26,27].

5. **Improving Data Integrity, Collection and Comparability to Achieve Strategic Goals**

Ensuring the EU Minimum Professional Standards for Midwives are aligned with Global Standards helps Member States to contribute to EU and global health initiatives relating to women’s health and equality [28,29]. It also ensures more accurate progress measurement between Member States, and better targeted strategic investments in improvement schemes.

The current European policy context provides a unique and timely policy window and opportunity to realise these benefits, where midwifery is increasingly recognised as contributing to broader women’s health and gender equality objectives. The European Parliament has called on the EU Commission to present an ambitious 2026–2030 Gender Equality Strategy, including the updating of the EU Directive on Professional Qualifications on midwifery, in alignment with international standards [30]. In addition, Members of the EU Parliament and over 50 Civil Society Organisations have issued recommendations for an EU Strategy for Women’s Health, highlighting the need for midwives to practise to their full scope, reflecting global standards [31]. Therefore, aligning the Directive would provide a more coherent framework through which progress towards these wider EU strategic goals can be supported and assessed across Member States.

6. **Maintaining the EU’s Global Leadership and Competitiveness**

The EU has historically been a global leader in midwifery, setting benchmarks in education, research, clinical practice, and professional regulation. Its comprehensive approach to sexual, reproductive, maternal, newborn and adolescent health has influenced international standards and guided reforms in many regions [32]. Countries around the world continue to view the EU as a model for excellence and innovation, particularly in integrating evidence-based practice, digital health, and interprofessional collaboration within midwifery education and care systems.

As the landscape of women’s health evolves—with rapid advancements in technology, data analytics, and femtech innovation—there is growing global competition in shaping the future of midwifery and maternal health. Aligning the Directive with the latest global standards and competencies will ensure that EU-trained midwives remain at the forefront of professional excellence, research leadership, and technological integration. This alignment will not only reinforce the EU’s position as a global reference point in midwifery, but will also enhance its competitiveness in emerging health sectors, stimulate cross-border collaboration, encourage the exchange of knowledge and good practices across regions, and contribute to stronger midwifery systems globally.

**Box 1. Policy recommendations for updating the EU Directive**

- Update Annex V of the Directive 2005/36/EC on Minimum Professional Qualifications - Midwife in alignment with ICM’s Global Standards and WHO guidelines.
- Ensure explicit recognition of midwives’ full scope of practice to promote safe, high-quality, and accountable care across the EU.
- Reflect regularly revised and evidence-based professional standards in the updated Directive.
- Strengthen policy coherence between EU legislation and internationally endorsed professional competencies.

## Conclusion

There is global and regional consensus that legislation relating to the midwifery profession should be aligned with ICM and WHO standards. This ensures quality, comparability, free movement of workforce, and will support the EU to achieve key strategic priorities. For this reason, it is critical that the update of the EU Directive on Minimum Professional Standards be aligned with the ICM Global Standards for Education and Regulation, which are in turn aligned with key WHO strategies and guidelines and most robust, recent scientific evidence. The revision of this Directive presents an opportunity to modernise midwifery regulation. It is important that policy coherence between international bodies and regional frameworks is in place. Aligning the Directive with ICM and WHO standards would strengthen European health systems and influence global health governance.
